# Pharmacy practice and injection use in community pharmacies in Pokhara city, Western Nepal

**DOI:** 10.1186/1472-6963-14-190

**Published:** 2014-04-28

**Authors:** Sudesh Gyawali, Devendra Singh Rathore, Kishor Adhikari, Pathiyil Ravi Shankar, Vikash Kumar KC, Suyog Basnet

**Affiliations:** 1 Department of Pharmacology, Manipal College of Medical Sciences, Pokhara, Nepal; 2 Department of Pharmacy, Rajasthan Pharmacy College, Jaipur, India; 3 Department of Pharmaceutical Sciences, School of Health and Allied Sciences, Pokhara University, Kaski, Nepal; 4 Department of Pharmacology, KIST Medical College, Lalitpur, Nepal; 5 Presently at Xavier University School of Medicine, Oranjestad, Aruba Kingdom of the Netherlands; 6 Department of Statistics, PN Multiple Campus, Pokhara, Nepal

**Keywords:** Community pharmacies, Developing countries, Health care waste, Injection disposal, Nepal, Safe injection practice, Sharps

## Abstract

**Background:**

Community pharmacies in Nepal serve as the first point of contact for the public with the health care system and provide many services, including administering injections. However, there is a general lack of documented information on pharmacy practice and injection use in these pharmacies. This study aims to provide information about pharmacy practice in terms of service and drug information sources, and injection use, including the disposal of used injection equipment.

**Methods:**

A mixed method, cross-sectional study was conducted in 54 community pharmacies in Pokhara city. Data was collected using a pre-tested, semi-structured questionnaire, and also by the direct observation of pharmacy premises. Interviews with pharmacy supervisors (proprietors) were also conducted to obtain additional information about certain points.

**Results:**

Interviews were carried out with 54 pharmacy supervisors/proprietors (47 males and 7 females) with a mean age and experience of 35.54 and 11.73 years, respectively. Approximately a half of the studied premises were operated by legally recognized pharmaceutical personnel, while the remainder was run by people who did not have the legal authority to operate pharmacies independently. About a quarter of pharmacies were providing services such as the administration of injections, wound dressing, and laboratory and consultation services in addition to medicine dispensing and counseling services. The ‘Current Index of Medical Specialties’ was the most commonly used source for drug information. Almost two-thirds of patients visiting the pharmacies were dispensed medicines without a prescription. Tetanus Toxoid, Depot-Medroxy Progesterone Acetate, and Diclofenac were the most commonly-used/administered injections. Most of the generated waste (including sharps) was disposed of in a municipal dump without adhering to the proper procedures for the disposal of hazardous waste.

**Conclusions:**

Community pharmacies in Pokhara offer a wide range of services including, but not limited to, drug dispensing, counseling, dressing of wounds, and administering injections. However, the lack of qualified staff and adequate infrastructure may be compromising the quality of the services offered. Therefore, the health authorities should take the necessary measures to upgrade the qualifications of the personnel and to improve the infrastructure for the sake of good pharmacy practice and the safer use of injections.

## Background

A community pharmacy is a unique combination of service and business, where pharmaceuticals are sold (business), and information is provided about the use of medicines and the prevention and treatment of diseases (services) [1]. Community pharmacies are in most cases the first point of contact used by millions of people seeking health care every day [2,3]. These community pharmacists could play a significant role in the self-management of minor illness by using over-the-counter (OTC) medicines, and hence improve the overall health of their communities [4,5]. In Nepal, community pharmacies are frequently visited by people who purchase medicine with or without a prescription, and who receive treatment (advice) for their illness from pharmacy supervisors (proprietors) or physicians visiting the pharmacy [5,6].

### The management of community pharmacies and their regulation in Nepal

In developed countries, community pharmacies are managed by qualified pharmacists. However, in developing countries such as Nepal, there are insufficient numbers of qualified and skilled pharmaceutical personnel [7]. In Nepal, according to the Drug Act of 1978, pharmacists, assistant pharmacists or "professionalists" (v*yabasai* in the Nepali language) can run a community pharmacy after registering the pharmacy with the Department of Drug Administration (DDA), which is the government body dealing with medicines and their related affairs [8]. Three levels of pharmacy personnel exist in Nepal: Pharmacists who have completed a 4-year Bachelor degree in pharmacy after 12 years of schooling; assistant pharmacists with a 3-year diploma in pharmacy after 10 years of schooling; and "professionalists," who only undertake a short orientation training course. Only pharmacists and assistant pharmacists can register with the Pharmacy Council of Nepal [9]. The 48-hour orientation training course was started in 1980, and was offered to individuals already involved in the pharmaceutical business [10]. Over the course of time, the duration of the training course was increased to 72 hours according to the feedback obtained from stakeholders [10,11]. Even after this increase in duration, the course was still found to be inadequate. It was recommended to discontinue the training and encourage those involved to pursue a diploma course in pharmacy [12]. This training has not been carried out for the past 12 years. All three categories of pharmaceutical personnel have equal authority to provide pharmaceutical services and run community pharmacies. Most of the registered pharmacies in Nepal are run by "professionalists" [7,10,13].

A few paramedical personnel, mostly health assistants (HA), and community medicine auxiliaries (CMA), also manage community pharmacies in Nepal. HAs and CMAs undergo basic medical training for 36 months and 18 months respectively, having already completed 10 years of schooling. They are trained to diagnose and treat common illnesses, and to refer patients for more specialized care if required [14,15]. They have the authority to treat patients suffering from minor illnesses and to prescribe a few selected medicines, but according to the Drug Act of 1978 [8], they do not have the legal authority to dispense medicines independently until they complete a pharmacy course or orientation training course.

### The use of injections

Injections are perceived as being one of the most powerful methods for curing diseases, and their use is popular in developing countries [16]. Injections satisfy the patients and yield "rewards" for the providers. Hence, their use is practiced by health providers in various sectors (e.g. traditional healers, paramedical persons, quacks), including the owners and staff of dispensaries/pharmacies [17]. The majority of injections used in developing countries are neither necessary, nor used safely [18].

Unsafe injection practice is a global challenge and considered to be a menace to public health in developing and transitional countries. The data indicates that the practice of reusing injection equipment without sterilizing it was the greatest (75%) in the South-East Asia Region, including Nepal [16]. It is estimated that the practice of providing injections unsafely accounts for infections such as 33% of all new cases of the Hepatitis B virus (HBV), 42% of all new cases of the Hepatitis C virus (HCV), and 2% of all new cases of the Human immunodeficiency virus (HIV) worldwide every year [18].

A safe injection "does no harm to the recipient, does not expose the health worker to any risk and does not result in waste that is dangerous for the community" [19]. Hence, the safe disposal of all the material involved in the administration of an injection is also important for promoting safe injection practices.

### The disposal of sharps (injections) and pharmaceutical wastes

Contaminated sharps (in particular, a used needle) pose a potential hazard to health, as they may cause injury, which in turn leads to the transmission of diseases [20]. Unfortunately, most of the therapeutic injection equipment (including used needles) used in developing countries are not disposed of safely [19,21]. The unsafe disposal of used injection equipment invites the risk of scavenging and resale. It also leads to needle-stick injuries (NSIs), thus exposing health care workers, waste handlers and the community to the risk of contracting blood-borne infections including HIV, HBV and HCV [17,20,22].

For the proper disposal of pharmaceutical waste, the role of pharmacists is well recognized in developed countries [23,24]. However, Nepal does not have official guidelines or practices to involve community pharmacies and pharmacists in the proper disposal of their pharmaceutical waste. In Nepal, the Solid Waste Management Act of 2011, the only act dealing with waste management, assigned legal and financial responsibility for the safe management of solid waste to the person or institution generating that waste [25].

Good pharmacy practice and the proper disposal of medical waste are key determinants of pharmacy services. However, there is a general lack of documented information on such practices in Nepal, which led to this current study being planned. This study aims to provide information about pharmacy practice in terms of the services provided, drug information sources, and the provision of injections, including the disposal of used injection equipment, in community pharmacies in Pokhara, Nepal.

## Methods

### Study area

The research was carried out from 15 September to 28 October 2012 in community pharmacies in Pokhara sub-metropolitan city, Western Nepal. Based on population size and infrastructure, cities in Nepal have been divided into three categories: Metropolitan, Sub-metropolitan and Municipality. Pokhara, one of the four sub-metropolitan cities, is the only sub-metropolitan city in Western Nepal, with a total population of approximately 256,000 people distributed over 18 wards [26]. This city was selected as the study area because it is the second biggest city in Nepal, and people from different socioeconomic strata live in the city. Furthermore, four researchers were originally from the city, which meant the study’s logistical operations were easier.

### Sampling

Out of the 350 pharmacies (including wholesale pharmacies and pharmacies attached to nursing homes and hospitals) in the city, 275 were community pharmacies. About 20% (54/275) out of these total pharmacies were considered for this study. Three pharmacies were selected from each of the 18 wards once the resource and time constraints on the part of the data collection teams had been taken into consideration, making a total of 54 pharmacies. Purposive sampling was carried out to select three pharmacies from each ward. The data collection team walked through different streets of a ward, and while passing along a street they randomly selected a pharmacy (but which met our inclusion criteria). The pharmacies were selected without considering their size, the number of people working in them, or the gender of the supervisor/proprietor. The next pharmacy was then selected from another street of the same ward.

### Study design and procedure

A mixed method, cross-sectional survey was carried out. The pharmacy supervisors (proprietors) of the selected pharmacies were approached for a face-to-face interview between 11 am and 4 pm local time. Those supervisors/proprietors present at the time of the researchers’ visit were requested to participate in the study. In most pharmacies, the data collection teams were able to meet the pharmacy supervisor/proprietors. In seven pharmacies, where the supervisors/proprietors were not present, the person immediately below the supervisor in the hierarchy was asked to participate. The study questionnaire was administered to the pharmacy supervisor, followed by an interview. The gender, experience, and qualifications of the participants were recorded.

Every interview was conducted in the Nepali language by two persons using an interview guide. One person asked the questions, while the second person observed the person being interviewed and took notes in English. The interviews were audio recorded with the permission of the interviewee. The pharmacy premises, medicine shelves, waste bins, and so on were observed after the interview using an observation check-list. The data collection was carried out by SG, AK, and SB, along with the help of the other pharmacists (as acknowledged) under the guidance of VKKC. Mr. Ajay Koirala (also acknowledged) helped us to establish contact with the respondents.

### Inclusion and exclusion criteria

Only medical shops (community pharmacies) on the territory of the sub-metropolitan city were included. Wholesale pharmacies, pharmacies attached to hospitals, and nursing homes in the city were excluded.

### Data collection tools

Three data collection tools were used in the study: A semi-structured questionnaire, an interview guide, and an observation checklist. These are described below.

#### The questionnaire

The semi-structured questionnaire was developed having taken into consideration this study’s objectives after a thorough review of the literature by the authors. The questionnaire was then circulated between pre-selected people working in the field, and feedback was gathered. This feedback was used to modify the questionnaire, which was pre-tested on a sample of 10 pharmacy supervisors/proprietors, and then any ambiguities in the questions or answers were addressed through consensus among the authors. The pre-tested questionnaire was then given to the participants. The data obtained from this pilot study was not used in the final analysis.

The questionnaire was divided into four parts: Part One collected demographic information from the participants; Part Two was about pharmacy services and dispensing practice; Part Three assessed injection practice; and Part Four dealt with waste disposal practice, including used injection equipment. The Additional file 1 shows the questionnaire used in this study.

#### The interview guide

Interviews with the supervisors/proprietors as key informants were conducted to obtain additional information about certain points mentioned in the questionnaire. An interview guide based on the questionnaire (shown in the Additional file 1) was used to provide a broad framework, but the interviewer had the freedom to ask additional questions when he felt there were areas to be explored beyond those mentioned in the interview guide. The respondents mentioned ‘Sangini’ (a popular injectable contraceptive in Nepal) training, so details of this training, and whether it was having any effect on their knowledge and skills of administering injections, were explored. The respondents were asked whether they provided any consultation and about the differences, if any, in the consultation provided by themselves and a physician visiting their pharmacy. In pharmacies where injections were administered, the pharmacy supervisor/proprietor was asked, "By whom and where are (venue) the injections administered?" "Where do you collect and dispose of the used syringes?" "Do you sell the used syringes?" "Do you charge separately for your injection service, or do you add the cost to the overall cost of the medicine?" In the disposal practice section, questions were asked to find out the reasons for the practices mentioned or observed. For example, if a respondent said they disposed of the waste in the municipal disposal system, he/she was asked, "Why do you dispose of your waste in the municipal waste system?" If the respondent said that they used a hospital disposal system for the disposal then he/she was asked for the name of the hospital and, "How and why do you dispose of your waste in this particular hospital disposal system?" The interviews were conducted on pharmacy premises. The interview was sometimes interrupted by patient visits or by some other personal reason on the part of the respondent. In these cases the interviews were resumed after the supervisor/proprietor was available. For each point (category) the respondent was allowed to freely express his/her opinion and each respondent was asked whether they had anything further to add before the interviewer proceeded to the next item on the interview guide. The questionnaire procedure was followed by an interview which lasted for 30–40 minutes. It took approximately 10 to 15 minutes to go through the questionnaire, and the interview lasted 25 to 30 minutes.

#### Observation checklist

The number of rooms in a pharmacy, the provision of a separate room for giving injections, the storage of injection equipment on medicine shelves, sharp waste (e.g. used syringes, cannula) being disposed of in waste collection bins, and giving advice or assistance in a counseling space/room were observed according to the checklist.

### Data analysis

For the statistical analysis of the quantitative data, the Statistical Package for Social Sciences (SPSS) version 17.0 for Windows was used. The qualitative data analysis was done using deductive content analysis [27,28]. The audio recordings of the interviews were transcribed by the interviewers. The interviews were conducted in the Nepali language and the transcript was also written in Nepali. The transcript was then translated into English by a person who was fluent in both languages and who was not associated with the study. The transcript of each interview was compared with the written notes and listened to by everyone involved to check for accuracy and content. The audio recording was listened to multiple times, compared to the transcript, and checked for errors. The data obtained was coded using a deductive approach, where the literature review and the interview guide were used to develop categories and subcategories for coding and analysis. Each transcript was read through several times to obtain a sense of the whole. Then all the words, sentences and paragraphs containing aspects connected to each other in terms of their content were coded in order to correspond with the predetermined categories [27,28]. Additional information provided by the interviewees was put into a different new category if required. Direct quotes were contextualized, rendered readable, and presented in the everyday language of the interviewees. These quotes were also translated from Nepali to English. The Consolidated Criteria for Reporting Qualitative Research (COREQ) checklist served as a framework for the qualitative research [29].

### Ethical issues

Ethical approval for the study was obtained from the Nepal Health Research Council (May 2012). Verbal informed consent was obtained from the supervisors/proprietors. Four pharmacy supervisors refused to participate in the study without giving any reason, so they were replaced with the nearest adjacent pharmacies from the respective wards.

## Results

Forty-seven (87%) male and 7 (13%) female supervisors/proprietors of community pharmacies, ranging in age from 20 to 57 years, and with a mean age of 35.54 years, participated in the study. The mean work experience of the respondents was 11.73 years. Table 1 shows the qualifications and training of the supervisors/proprietors of the community pharmacies. According to Nepal government law, only twenty-nine pharmacy supervisors (53.7%) [1 pharmacist, 12 assistant pharmacists and 16 "professionalists"] were legally entitled to run a pharmacy independently. The remaining pharmacy supervisors had neither a degree in pharmacy, nor had they completed orientation training, and were therefore not legally entitled to run a pharmacy independently. Out of 20 CMAs, only 3 (who had completed the orientation training) were qualified to provide pharmacy services.

**Table 1:** Qualifications of the supervisors/proprietors along with the training received

**Qualification**	**Training**				
	**No training**	**Orientation**	**Sangini**	**Other**	**Total (%), N = 54**
Diploma in Pharmacy	8	0	2	2	12 (22.2)
Bachelor in Pharmacy	1	0	0	0	1 (1.9)
Community medicine auxiliaries (CMA)	11	3	5	1	20 (37.0)
Health assistant	1	1	0	6	8 (14.8)
Nursing	0	1	1	0	2 (3.7)
Other ("professionalist")	0	11	0	0	11 (20.4)

All the respondents were asked for any training they had received in administering injections or in pharmacy practice; 8 respondents mentioned "Sangini" training (Table 1) and gave information about this training. Sangini (meaning "lady friend" in Nepali) training is a two-day training course offered by the Nepal CRS Company, which markets a popular injectable contraceptive in Nepal in association with a non-governmental organization (NGO). This training provides in-depth information about Sangini (Depot-Medroxy Progesterone Acetate, DMPA), a popular injectable contraceptive brand marketed by the company, along with other family planning methods [30]. Following the training course, each participant was provided with a red, plastic bucket for the collection of used Sangini auto-disable syringes in their pharmacies (Sangini outlets). These buckets were reused after emptying.

### Services offered by the pharmacies

The pharmacies selected for this study provided their services for between 5 and 15 hours a day. Most (44 out of 54) remained open for 12 hours or more daily (a mean of 12.24 hours). On average each premises had 2 working staff, but in some cases we found 1, or even up to 5. This number also included pharmacy supervisors (mostly the owners of a pharmacy).

Table 2 shows the diversified services offered by the community pharmacies. Medicine dispensing and counseling were a part of pharmacy practice, while other services may not be considered as such. Twenty-six pharmacies were providing physician consultation services, but only seven were providing this service on a regular basis. A regular physician consultation service provided by a pharmacy was for a mean period of 4.14 hours daily. Those supervisors said that they provided primary health care to patients, but when patients needed or demanded a specialist consultation, they were advised to visit the pharmacy during the scheduled time of a physician’s visit (generally during the morning and/or evening time).

**Table 2:** Services offered by the pharmacies

**Name of services**	**Frequency (%) N = 54**
Medicine dispensing & counseling, wound dressing and injection administration	20 (37.03)
Medicine dispensing & counseling, wound dressing, injection administration, lab facility and physician consultation	14 (25.93)
Medicine dispensing & counseling, wound dressing, injection administration and physician consultation	11 (20.37)
Medicine dispensing & counseling, wound dressing, injection administration and lab facility	4 (7.41)
Medicine dispensing and counseling only	4 (7.41)
Medicine dispensing & counseling, and physician consultation	1 (1.85)

### Dispensing practice

All the pharmacy supervisors who took part in this study said that they provided consultations (in minor cases by themselves and in major cases by a physician visiting the pharmacy) and dispensed medicines. Fever, gastritis, coughs and colds (flu-like symptoms), diarrhea, wounds and injuries, and pain were the most common conditions for which patients consulted the supervisors.

Out of the 54 pharmacy supervisors, 34 (55.6%) dispensed full courses of medicine without a prescription and attempted to treat patients themselves, while 18 (33.3%) and 5 (9.3%) pharmacy supervisors disclosed that they dispensed only over the counter (OTC) medicines and advised patients to consult a doctor when patients visited them for treatment. One pharmacy supervisor said that any consultation depended on the condition of the patient’s health and patient expectations. He said, "*If patient is not very serious and willing for few medications then I provide him/her medication. However, if the patient is serious and/or willing to consult doctor then I help him/her for the appointment with the doctor*".

The mean number of prescriptions filled or refilled was 9.41 per day per pharmacy, and the average daily number of patients visiting a pharmacy without a prescription was 29 per pharmacy. The average number of patients visiting a pharmacy per day to receive or repeat their prescription was much less (32.7%) when compared to patients visiting a pharmacy to obtain medicine without a prescription. Thirty pharmacy supervisors (mostly paramedicals) said that they helped patients by providing consultation services and dispensing medicine. One of the supervisors with CMA qualifications said, "*People believe me and come for my consultation service. I have been trained for medicine use then why I should not help the people?..... when I cannot handle the case then I refer them to physician.*" Forty-five (83.3%) supervisors said that they dispensed full courses of medicine (including antibiotics, and injections if required) to their patients without a prescription. Similarly, 5 (9.3%) supervisors stated that they did not dispense any medicine, but would ask their patients to consult a doctor. Three (5.6%) supervisors claimed that they dispensed only OTC medicines without a prescription. In one pharmacy where the supervisor claimed to dispense only OTC medicines without a prescription, we observed during our visit to that pharmacy, that paracetamol tablets were dispensed along with ciprofloxacin (antimicrobial, not an OTC) tablets for 5 days without a prescription to a patient complaining of running a fever for a couple of days.

The most commonly-dispensed medicines without a prescription by the pharmacies were non-steroidal anti-inflammatory drugs (NSAIDs), antimicrobials (including metronidazole), anti-cold remedies (for flu-like symptoms), gastric acid secretion blockers (H2 blockers/Proton pump inhibitors), hormonal contraceptives, and cough preparations.

Most of the pharmacies [40 (74.1%)] checked manually for out-of-date drugs on their premises, 12 (22.2%) did so at the time of dispensing, and only 2 (3.7%) pharmacies used computer software to check the status of any out-of-date drugs in their pharmacy.

The pharmacy supervisors depended on one or more source(s) for drug information. Table 3 shows the various sources (in different combinations) they used for drug-related information. The "Current Index of Medical Specialties (CIMS)" was one of the most important sources of information on drugs, and which was used along with other sources.

**Table 3:** Sources of drug information

**Sources of drug information**	**Frequency (%) N = 54**
Medical index (CIMS/MIMS etc.)	19 (35.19)
Medical index and Medical representatives	9 (16.67)
Medical index and product leaflets	7 (12.96)
Medical index, Medical representatives and product leaflets	5 (9.26)
Internet and Medical index	4 (7.41)
Product leaflets	4 (7.41)
Internet, Medical index and Medical representatives	3 (5.55)
Medical representatives	3 (5.55)

### Infrastructure of the counseling rooms

Only 25 (46.30%) pharmacies had a separate counseling room for giving advice. Although the supervisors claimed that the rooms were used for patients counseling, they did not contain any aids such as books, dummy medications, or models of internal organs. All of the rooms (except 1) contained a bed, a table, chairs, and stools, along with books such as CIMS/MIMS (the Monthly Index of Medical Specialties), and a few posters on the walls. The rooms looked more like physician consultation rooms than counseling rooms. However, one community pharmacy managed by a registered pharmacist had a counseling room with a computer, books (the American Hospital Formulary Service and other reference books), posters, pamphlets (about diabetes mellitus, hypertension, and medicines), and equipment. Information about the book editions, or whether the books were current or not, was not included in this study.

### The use of injections

Forty-nine (90.7%) pharmacies dispensed and administered injectable medicines. Almost all of the dispensed injectables were administered at the pharmacy itself. In one instance, during the study, a patient with some medicine visited a pharmacy supervisor to receive that medicine as an injection (i.m.). The patient reported that the medicine had been prescribed by a doctor at a tertiary care hospital at the time of discharge and was purchased from the hospital pharmacy. In a majority of pharmacies, the injections were administered by the supervisor him/herself in a separate room. The patient counseling rooms were also used to administer injections, although they were not well-equipped enough for an injection procedure as they lacked safety boxes, a separate injection preparation area, and running water.

Tetanus Toxoid, DMPA (an injectable contraceptive), Diclofenac, intravenous fluids (IVFs), and anti-ulcer medicines (H2 blockers/Proton pump inhibitors) were the five most commonly dispensed (administered) injections by the community pharmacy (n = 49) supervisors/proprietors. Some "counseling" rooms contained a bed which was used to administer IVFs.

Long acting contraceptive (effective for 3 months) were the most commonly administered injections. The supervisors reported that even women from rural areas were visiting their pharmacies for injectable contraceptives. They reported that, although these injections were available at government health care facilities free of charge, those rural women preferred a pharmacy because they wanted to guarantee total secrecy about the injection event and do not want that their families to know about the injection. One of the representative statement was, "*……….the rural women come all the way to me for SANGINI* (DMPA). *They want to keep it secret as most of their husbands are abroad for work* (foreign employment). *……….*"

Out of the 5 pharmacies where supervisors claimed that neither injection equipment were dispensed nor injections administered, the medicine shelves in 2 of the pharmacies contained injectable medicines and equipment. Out of the 49 pharmacy supervisors who reported administering injections in their pharmacies, 38 (77.6%) of them charged for their injection services, while 11 (22.4%) claimed to provide the service free of charge. A service charge was included in the price of the medicines so patients may not have been aware of the charges they had paid.

### The practice of waste disposal

Table 4 shows the different methods used to dispose of various types of health care waste generated by pharmacies. Disposing of waste in municipal dumps without following the proper procedures for the disposal of hazardous waste, burning waste in an open area, and disposing of waste in a hospital’s waste disposal system were the most common methods used by community pharmacies.

**Table 4:** Methods of disposal of pharmacy wastes

**Method of disposal**	**Number of pharmacy (%)**			
	**Non-infectious waste, N = 54**	**Non-returnable expired Medicine, N = 54**	**Sharp waste, N = 53**	**Disposable syringe with needle, N = 51**
Incineration	5 (9.3)	1 (1.9)	2 (3.8)	1 (2)
Burying	3 (5.6)	5 (9.3)	13 (24.5)	3 (5.9)
Burning in the open air	17 (31.5)	11 (20.4)	6 (11.3)	7 (13.7)
Municipal dump	27 (50.0)	34 (63.0)	25 (47.2)	25 (49)
Disposal in hospital waste system	1 (1.9)	1 (1.9)	7 (13.2)	9 (17.6)
Selling to scrap purchasers (Kabadis)	-	-	-	6 (11.8)
Other	1 (1.9)	2 (3.7)	-	-

The used syringes in the pharmacies were either collected along with the needles (without destroying them) in a red (Sangini) bucket, or in an open container. The collected waste was either disposed of in a municipal waste disposal system or disposed of with no safety precautions (including open-air burning). A 52-year-old male "professionalist" with a CMA qualification said, *"… there is no space for disposal of waste so I collect the used syringes in that box* (Open carton) *and take it to my home to burn the syringes…… other waste is thrown in municipality waste truck……".*

**Figure 1:**
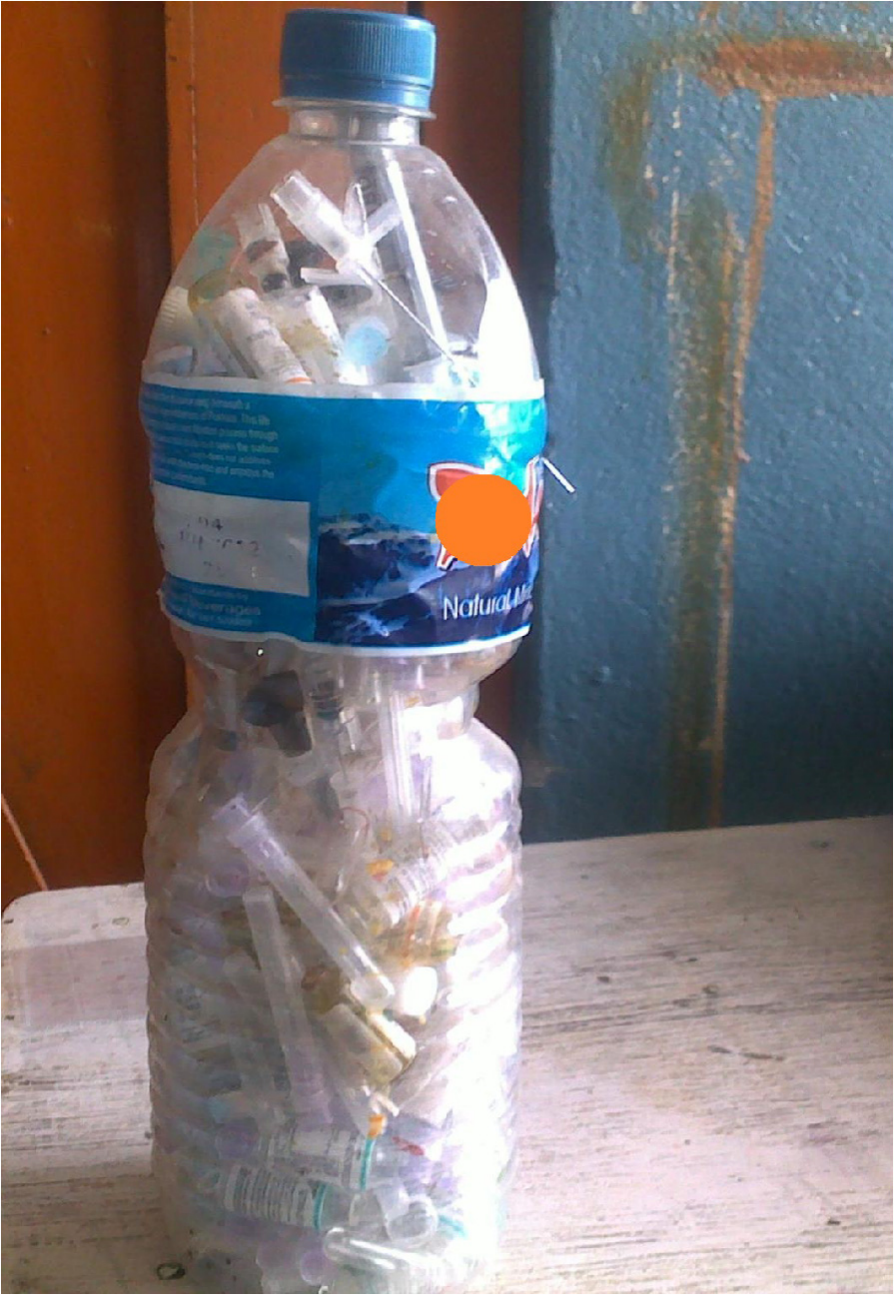
**A mineral water bottle used as a sharps container.** The bottle used was not puncture proof.

**Figure 2:**
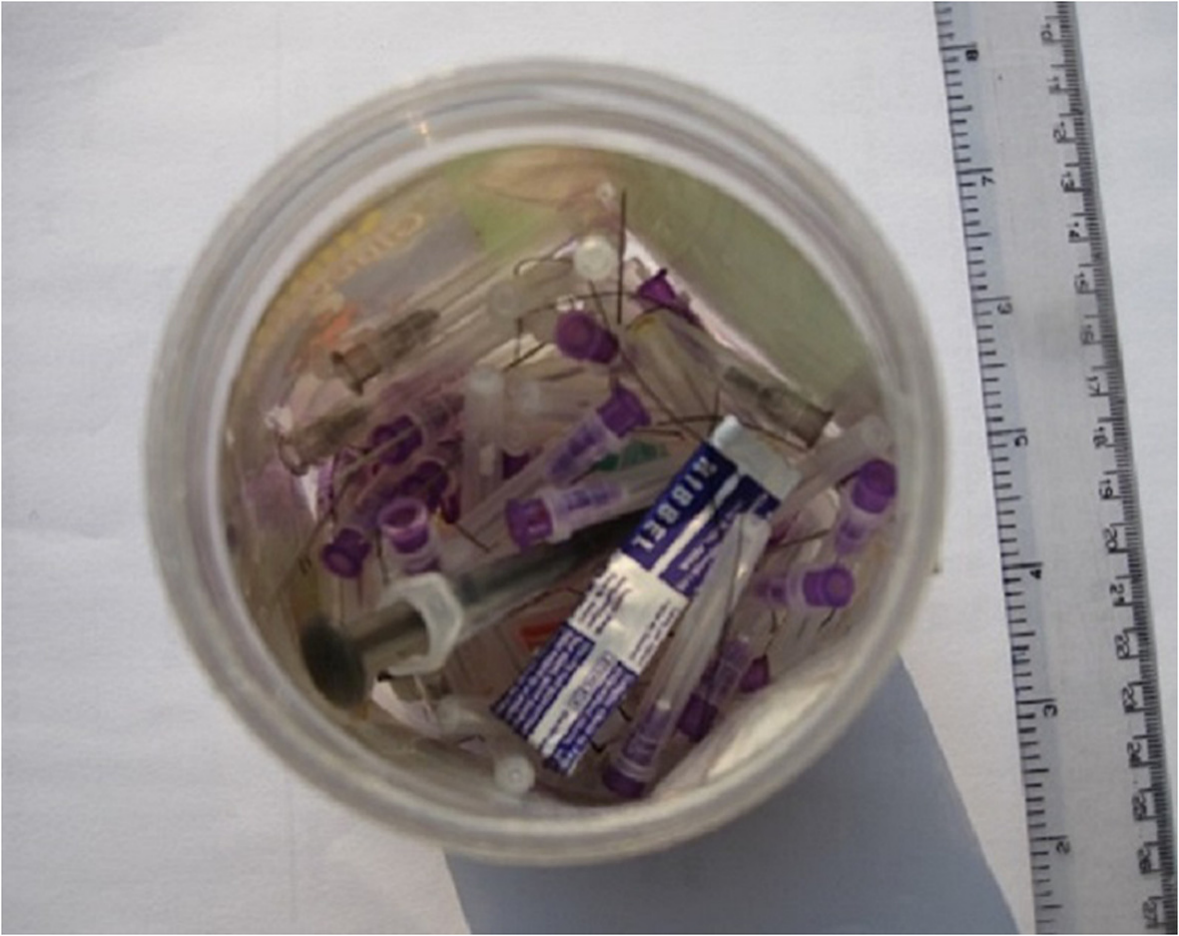
**A small hard plastic container used as a sharps container.** The container used had a wide opening and contained recapped used needles.

Few pharmacy supervisors reported that they disposed of their used syringes (along with the needles) and other sharp waste separately in a nearby or associated (with the supervisor or visiting physician) hospital, but no separate containers were found to be in use during our observational study. During the survey it was also found that 3 pharmacy supervisors were collecting needles and sharps in self-designed containers; a mineral water bottle (Figure 1) or a hard plastic container with a wide opening (Figure 2). The bottle was not puncture proof, placing handlers at risk of NSI. The hard plastic container was small with a wide opening. The recapped used needles, suture needles and other sharps were collected in that container. The pharmacy supervisors were looking for a proper method for the disposal of their waste. They said that they would ask the physician visiting their pharmacy to help them in disposing of the collected sharp waste in the waste collection system of the tertiary care hospital with which the physician was associated, or as a last resort they would have to dispose of their waste in the municipal waste system.

Twenty-seven pharmacy supervisors (Table 4) said that they had to hide their waste and sometimes bribe the municipal waste collectors to dispose of their waste in the municipal collecting unit because the waste collectors were instructed by the authorities not to collect hazardous medical waste. One of the statements was,*"……since there is no better option left for us…… to dispose of the waste, we hide the sharps (including syringes with needles) and infectious waste in common* (non-infectious) *waste and drop in the municipality waste collection system……"*.

Similarly, a 50-year-old male pharmacy supervisor/proprietor said, *"Needle destroyer has been sent for repair……. so I throw* [*used syringes*] *in municipality waste….. I give Rs. 50 [on every encounter] to the driver of municipality waste collecting vehicle so he* (the driver) *carries otherwise he would have refused to collect".*

**Figure 3:**
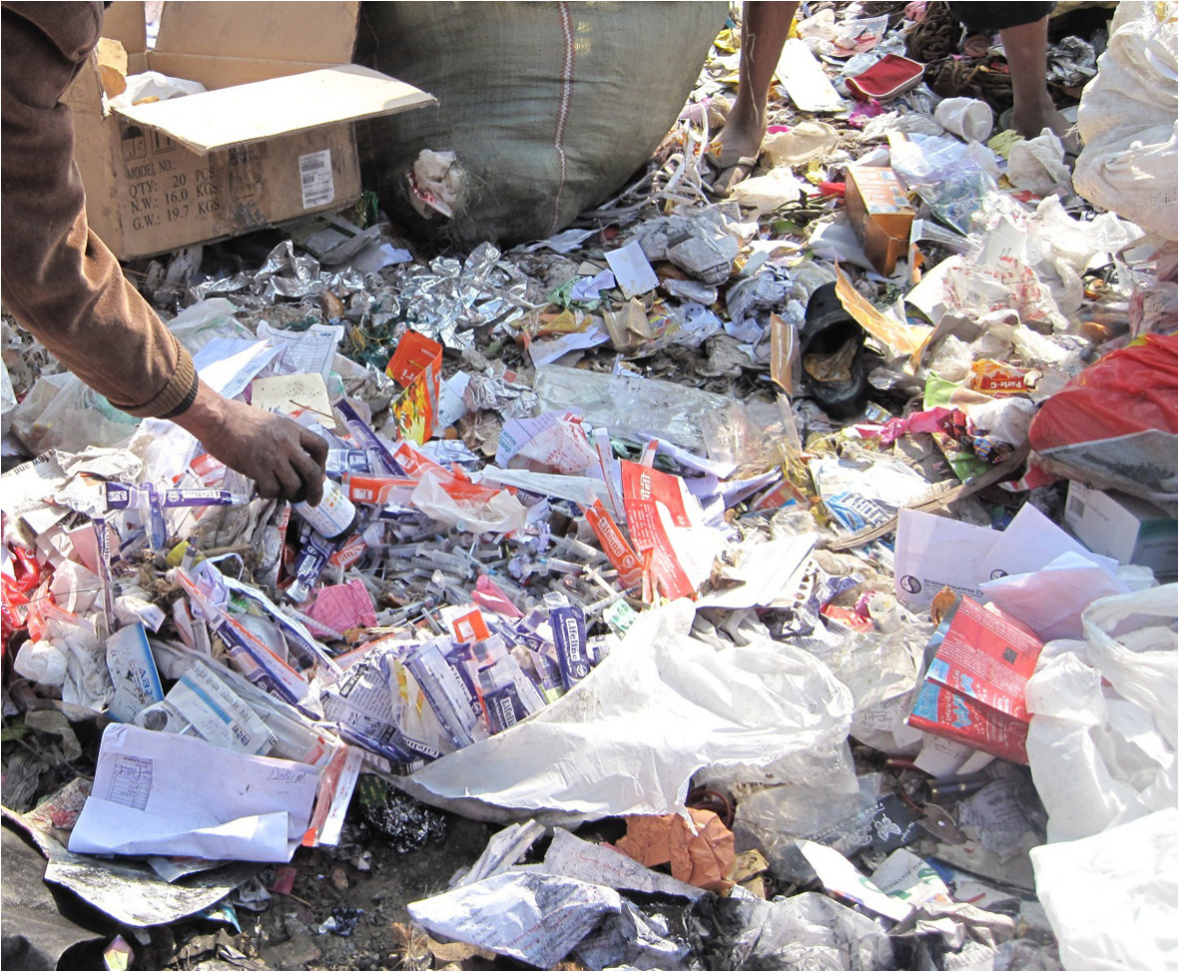
**Package of used syringes found by waste handlers at a municipal dump while sorting and scavenging for waste items for sale.** The waste handlers were doing their job in an unsafe manner without wearing any protective clothing.

The city’s land-fill site was also visited during the study and used disposable syringes were commonly observed among the municipal waste. The waste handlers sort the waste in order to scavenge items that can be sold (reused) in the market. The sorting was done by waste handlers who were wearing no protective materials such as gloves, masks, boots, aprons, and so on (Figure 3).

Most supervisors said that scrap purchasers (*Kabadis)* visited their pharmacies in search of used syringes to buy, but only 6 supervisors admitted selling used syringes to them. They believed that they were forced to do so. A 45-year-old female "professionalist" said, *"…… We don’t have space to burn the* [used] *syringes and municipality authority neither accept the syringes nor give an alternative disposal solution so we are compelled to do so…… by selling the syringes we are saving the environment* [by recycling the plastic] *and getting* [making] *some money as well".*

## Discussion

### Pharmacy personnel and their services

This study revealed that the majority of pharmacies were run by "professionalists," as reported by other studies [6,13]. However, the proportion of community pharmacies run by assistant pharmacists was higher compared to a previous study conducted in the 6 main cities of Nepal [13]. This might be due to the increasing number of graduating assistant pharmacists in Nepal. Although the number of graduating pharmacists is increasing, the number of registered pharmacists working in community pharmacies is still low [9]. This might be due to the discouraging provision in the Drug Act of Nepal, which permits pharmacists, assistant pharmacists and "professionalists" to run pharmacies without taking into consideration the differences in their qualifications. The government should address the problem and encourage pharmacists to choose community pharmacy practice as a career. One approach might be to categorize the pharmacies and give more authority to pharmacists as compared to others.

About half of the community pharmacies studied were run by paramedicals who had neither a pharmacy degree nor undergone orientation training. These supervisors were neither qualified, nor legally entitled to independently operate a pharmacy, but were qualified for treating minor illnesses and administering injections. As 30 supervisors with paramedical degrees said that they provided consultations to patients and dispensed medicines themselves, this might have contributed to the higher number of patients visiting community pharmacies without prescriptions. Even though more than 48% of pharmacies were offering physician consultation services, more than half of the dispensers admitted treating patients themselves before referring them to a physician. A study conducted by Shankar et al. in Pokhara sub-metropolitan city also showed comparable results, with 38.8% of respondents from the city depending on community pharmacies for their medical needs. Pharmacies were preferred by the local people, as they could obtain not only allopathic medicines, but also ayurvedic (traditional) medicines with or without a prescription [6]. Furthermore, purchasing medicines directly from pharmacies may have been cheaper, as patients did not have to pay physician consultation fees or medical investigation expenses [5]. These pharmacies also provided other services such as injections administration, laboratory investigations and wound dressing apart from dispensing, counseling, and physician consultation facilities. These might also be reasons for their preference by the public.

All the dispensers claimed to counsel their patients, but only about half of them had a separate space set aside for counseling purposes. A separate space will help to make patients more comfortable. Patients may hesitate to ask questions or share their understanding (about medicine and disease) publicly. A quiet and separate space makes patients feel that their health problems will remain confidential.

To obtain information about medicines, the pharmacy supervisors depended on non-objective sources such as CIMS and MIMS, as well as medical representatives from pharmaceutical companies. A similar situation was seen in a previous study [13]. Poor medical advice leads to poor compliance with the therapy, and ultimately to a poor therapeutic outcome.

In developed countries, only a qualified pharmacist is allowed to run a community pharmacy, and the pharmacists have to undertake specified annual continuing professional education [4]. The pharmacist accepts challenges and provides services as a health advisor, so their role is highly esteemed and acknowledged by general practitioners in developed countries such as Australia, the USA, and the UK among others. Surprisingly, many general practitioners in developing countries do not regard pharmacists as potential members of a health care team [3]. A lack of competence (less qualified pharmaceutical personnel) and the provision of a variety of services may be hindering factors towards the provision of quality services. In addition to pharmaceutical services they also provide diagnostics, dressings, injections, physician consultations, and so on.

To reduce the considerable medication-related morbidity and mortality, inter-professional collaboration between doctors and pharmacists is advocated in developed countries [31]. In our study, about half of the pharmacies were providing a physician consultation service, so there is a high possibility for inter-professional collaboration between doctors and pharmacy supervisors. Fresh pharmacy graduates in Nepal should choose community pharmacy practice as a career and use this opportunity to take direct responsibility for their individual patients’ medication needs. This type of patient-centered care ensures safety, effectiveness and adherence to therapy, which in turn all contribute to better treatment outcomes. The adoption of these roles would also help to clear the tarnished reputation of pharmacists as drug dispensers [31].

### The use of injections

Only 30 (55.56%) pharmacy supervisors with CMA, HA, and nursing qualifications were trained to administer injections, but almost 50 (92.6%) supervisors were administering injections to their patients. This indicates that a significant number of injections were also administered by "professionalists" who were not formally trained for this procedure. Furthermore, as injection practice was a sensitive issue, this question was not always answered properly. This might be the reason for proprietors having injectable medicines on their medicine shelves, but their stating that they neither dispensed nor administered injections in response to our queries. In Nepal there is no clear guideline, policy, or qualification for injection providers [32].

Important and useful injectable medicines such as Tetanus Toxoid, contraceptive injections (DMPA), and Diclofenac were commonly administered in the pharmacies. Therefore, it may not be wise to ban the administration of these injections for this category of health workers (who might be untrained) because people may either not obtain the necessary injections, or they may visit quacks for the same [17]. Both could have negative consequences for public health. Therefore, personnel working in community pharmacies should be trained and given the authority to provide only a few useful injections safely and rationally. Nevertheless, care has to be taken as this could be considered as tacit approval for these personnel to administer all types of injection. Further research is required to identify the vital (life-saving) injections which they would provide. Policy to regulate and promote their rational use while considering all aspects of safe injection practice [17] is also necessary.

Even though injectable contraceptives were available free of charge at government health care facilities, women preferred to receive their injections from pharmacies. One of the most convincing reasons, as reported by the supervisors, may have been secrecy. The women did not want their families to find out about their injection. One possible explanation could be that they were having an extramarital affair, so they required contraceptives even though their husbands were abroad. This type of social problem has been reported in the newspapers [33,34].

### Waste disposal practice

Waste disposal practice in most community pharmacies was not satisfactory. They lacked the space and infrastructure for the proper disposal of their medical waste. As open-air burning of health care waste carries risk to staff, communities, and the environment, it is not recommended by the WHO [35]. Unfortunately, the practice of open-air burning was evident in our study. The disposal of waste by community pharmacies in the municipal waste collection system in a haphazard and unsafe manner, and even by bribing the relevant authorities, was as high as 63% in our study. In situations where no other options for the safe disposal of health care waste are available, disposal in sanitary land-fills or municipal land-fill sites while taking precautions could be an acceptable option only for pharmaceutical waste [20,35]. The disposal of small quantities of pharmaceutical waste, while taking the necessary precautions, in a municipal waste disposal system is also recommended by the US-Food and Drug Administration [36]. This method may be regarded as a provisional measure, which should be improved upon to achieve ideal disposal methods in the future [37].

As in South Asian [38,39] and other countries [40], the selling of used plastic (disposable) syringes to scrap purchasers (*Kabadis*) was also evident in our study. Such practices might lead to NSIs among waste pickers and can lead to the illegal repackaging of syringes for reuse in hospitals and clinics [39,40]. An outbreak of hepatitis B due to the use of illegal repacked syringes was reported in India [40]. The resale of used disposable syringes and other injection equipment after washing and repackaging without sterilization was also commonly reported in South East Asian countries such as Pakistan, India, and so on [40]. The lack of a legally authorized body to regulate (check) the quality of injection equipment manufactured in and/or imported by Nepal [14,32] makes the improper disposal of needles a serious issue requiring urgent attention.

Used syringes (without needle recapping) and other sharps should be collected in a specially designed puncture and leak proof sealable box [17,20]. The use of safety boxes is important for ensuring the safety of the injection providers [18]. The use of open cardboard boxes or red buckets may not be a good alternative to safety boxes. Furthermore, safety boxes filled with used syringes should be incinerated or destroyed using other methods [19]. They should not be re-used, but in our study the sharp collection containers were in fact reused.

In this study, it was found that 3 pharmacy supervisors collected needles (after recapping) and other sharps in a self-designated container such as a mineral water bottle (not puncture proof) or a container with a wide opening. They believed that this practice was good. This indicated a lack of proper knowledge about the importance and methods of waste management. A number of studies have indicated that educating and creating awareness among health professionals about health care waste leads to safer disposal [20,41-43]. In view of this, training to improve skill and awareness is required.

### Epilogue

Policies and plans for the safe management of health care waste and for addressing the issue of waste generated by these pharmacies are urgently required. It may not be financially viable for each pharmacy to have an incinerator. Therefore, a communal treatment facility for health care waste managed by the municipal authorities could be a more cost-effective option. According to Section 3 of the Solid Waste Management Act 2011, the installation of a communal incinerator, a system for the collection of health care waste (including sharps), and transportation could be organized by the municipality, for which the health care facilities (including community pharmacies) could be charged [25]. This type of common treatment facility shared by larger and smaller health care facilities was found to be cost-effective for operations and maintenance in Indonesia [42].

### Limitations

The descriptive study addressed many areas of pharmacy practice and injection use. Thus, in-depth analysis may be lacking in some areas, such as the quality of services offered by the pharmacies. Similarly, this study focused on the sources of the drug information, but not on the quality of the sources themselves. Due to the sensitive nature of some of the questions, all the answers given by the community pharmacy supervisors may not have been consistent with actual practice. Therefore, observations and interviews were also conducted. Repeat interviews with the interviewees and checking of the transcripts by the respective interviewees could not be done because of time and resource constraints, although the respondents were asked whether they had anything further to add about a point before going onto the next item on the interview guide. As with all survey questionnaires, participant recall bias may have occurred.

## Conclusion

Community pharmacies in Pokhara offer a wide range of services, including, but not limited to, drug dispensing, counseling, dressing of wounds, and administration of injections. However, a lack of qualified staff and adequate infrastructure may compromise the quality of these services. Further in-depth research is required. There is also a need for policies and plans to develop and encourage qualified manpower in community pharmacies and to improve pharmacy practice. The possibility for inter-professional collaboration between doctors and pharmacists for better patient care could be explored. Considering the qualifications of the provider, under-equipped injection rooms and the disposal of the used syringes, it can be concluded that there are plenty of grey areas which should be improved on to make the use of injections safer. Proper training for the personnel on the administration of a few useful injections, restricting the administration of other unnecessary injections, and addressing the issue of the disposal of used equipment are required to make injection practice safer.

## Competing interest

All the authors declare that they have no competing interests.

## Authors’ contributions

SG, DSR and PRS conceived and designed the study. SG, DSR, PRS, VKKC and KA finalized the methodology and tools used. SG, KA and SB collected the data. SG, KA, SB and VKKC analyzed the data and drafted the manuscript. All the authors made significant contributions to writing the manuscript and reviewing the literature. The final manuscript has been read and approved by all the authors.

## Pre-publication history

The pre-publication history for this paper can be accessed here:

http://www.biomedcentral.com/1472-6963/14/190/prepub

## Supplementary Material

Additional file 1 Questionnaire used in the study. Click here for file
