# Supplementary material for: Pharmacy practice and injection use in community pharmacies in Pokhara city, Western Nepal
Source: BMC Health Serv Res. 2014 Apr 28;14:190. doi: 10.1186/1472-6963-14-190 (PMC4101856; doi:10.1186/1472-6963-14-190)
Supplement: Additional file 1 — Questionnaire used in the study. [file 1472-6963-14-190-S1.docx]

**QUESTIONNAIRE**

**Demographic information of the in-charge of Pharmacy (Medical Shop)**

Age :

Sex :

Qualification :

Experience :

Training (if any):

Working hours (person):

Number of staff working in Pharmacy:

**Pharmacy Services**

1. Services provided by pharmacy:

a) Dispensing b) Counseling (separate room YES/NO)

1. Dressing d) Lab testing

e) injection administration f) physician consultancy (on call/regular)

1. others…………………………………….
2. **If regular consultancy is provided,** Average working hour of doctor:

**Dispensing Practice**

1. How many prescriptions do you refill/fill per day (Average)? …………………
2. Do patients come to consult you for treatment? **( Yes/ No)**

**If yes to question number 2,**

i) What are the 5 most common conditions for which they consult you?

1. …………………………………
2. …………………………………
3. …………………………………
4. …………………………………
5. …………………………………

ii) What do you suggest to them?

1. Counsel them to consult a doctor
2. Treat them with full course of medicine
3. Dispense some OTC drugs for short period (1-2 days) and ask them to see a doctor
4. Other ………………………………..
5. How many patients visit your pharmacy to obtain drugs without a prescription? ……….
6. **(Pertaining question number 3)** What do you do with them?
7. Dispense Medicine b. Dispense OTC drug only
8. Counsel them to see doctor d. Other…………………
9. Can you please tell me 5 most commonly dispensed drug without prescription?

i) …………………………… ii) ………………………… iii) …………………………

iv) …………………………… v) …………………………

1. How many parenteral (Injections) preparations do you dispense per day (Average)? ……
2. Can you name five most commonly dispensed parenteral (Injections) preparations?
3. ……………………………………..
4. …………………………………….
5. …………………………………….
6. …………………………………….
7. …………………………………….
8. Do you administer the injection? **(Yes/ No)**
9. If yes to question no. 8,
10. How many injection per day …………………………….
11. Can you please name 3 injectable drug you administer most………….
12. How much do you charge (Service) for administrating injection……..
13. How do you check for expired drug in your store?
14. Manually
15. Electronic software
16. During dispensing
17. Other……………………..
18. What is your source of drug information?
    1. Internet
    2. CIMS/MIMS
    3. Medical Representative
    4. Product leaflets
    5. Other………..

**Disposal**

1. How do you dispose waste produced from your pharmacy?
2. Incineration
3. Burying
4. Burning in open place
5. Municipality waste vehicle
6. Other………….
7. How do you dispose expire medicine that are not returned back?
8. Incineration
9. Burying
10. Burning in open place
11. Municipality waste vehicle
12. Other (please specify) …………………………………
13. How do you dispose Sharp waste?
    1. Incineration
    2. Burying
    3. Burning in open place
    4. Municipality waste vehicle
    5. Other (please specify) ………………………………………
14. What do you do with used disposable syringes?
    1. Reuse e. Sell to Kabadi
    2. Incineration f. Burying
    3. Burning in open place g. Other…………………………
    4. Municipality waste vehicle
